# Institutional suicide as anomie: decedents speak out for work-related suicides through a Durkheimian exploration of suicide notes in a context without institutional responsibilization for suicide prevention

**DOI:** 10.3389/fsoc.2024.1309119

**Published:** 2024-03-04

**Authors:** Mustafa F. Ozbilgin, Cihat Erbil, Orkun Demirbağ, Nur Gündoğdu, Kübra Şimşek Demirbağ

**Affiliations:** ^1^Brunel Business School, Brunel University London, London, United Kingdom; ^2^Department of Business Administration, Ankara Haci Bayram Veli University, Ankara, Türkiye; ^3^Department of Business Administration, Gümüşhane University, Gümüşhane, Türkiye; ^4^Department of Accounting, Birmingham Business School, University of Birmingham, Birmingham, United Kingdom; ^5^Department of Management Information Systems, Gümüşhane University, Gümüşhane, Türkiye

**Keywords:** institutional suicide, anomie, recognition, misrecognition, institutional responsibilization

## Abstract

**Introduction:**

Drawing on Durkheim’s historical theorization of suicide, we extend his concept of anomic suicide, which is suicide due to a lack of social regulation, to introduce the concept of institutional suicide. We define institutional suicide as suicide due to the absence or decline of institutional policies, practices, and discourses for prevention. In this study, we explore the mechanisms for institutional suicides based on suicide notes Turkey, in a context without institutional responsibilization for prevention. Turkey provides a significant context for studying institutional suicides as policies, practices, and discourses for suicide prevention have been declining for some decades.

**Methods:**

Drawing on publically available suicide notes and narratives in Turkish media outlets, we analyze 17 suicide notes and responses from their institutions of work and friends, family, and colleagues.

**Findings and Discussion:**

We identify two mechanisms that lead to institutional suicides: (1) dehumanization due to lack of recognition and (2) misrecognition through a devaluation of potential. We extend the theory of anomie to institutional settings and offer social policy suggestions to improve institutional responses based on co-design based on suicide notes to prevent institutional suicides and call for institutional responsibilization for preventing work-related suicides.

## Introduction

Suicide is one of the three pillars of deaths of despair (Case and Deaton, 2022) and a major global health problem (WHO, 2021). In the mainstream literature, suicide is an individual act (deliberate and voluntary) of attempting to end one’s own life (Chandler et al., 2022). However, such individual-level perspectives often overlook the impact of concepts such as psychological resilience, which may shift the focus from institutional responsibilities to individual issues in addressing suicide. They focus academic attention on psychiatric disorders and other individual risk factors without concentrating on the surrounding context that prepares grounds for suicide (White and Kral, 2014; Waters, 2017a,b). Suicide has varied meanings in different cultures (Snowdon, 2018). In some Western cultures, suicide is a sinful individual act which should be prevented (Minois, 2001). In Eastern cultures, suicide is a form of sacrifice or an honorable consequence of paying for one’s mistakes (Kawanishi, 2008). In certain societies, suicide is upheld as a commendable action, while in others, it is considered an undesirable choice, prevented with legal and social measures (Headley, 2023). Despite cultural differences, national laws view suicides at work as individual choices rather than an outcome of inadequate institutional measures for prevention (Waters and Palmer, 2022). Similarly, cultural and social framing of suicide borrow elements from religious traditions and focus on individual choice, even in cases of suicides in organizations. This type of framing is surprising considering that suicides peak in times of institutional decline, scandals and collapse and the decline of the economy and politics (Huey and McNulty, 2005; Norström and Grönqvist, 2015; Oyesanya et al., 2015). For example, preceding the 2007–2008 economic crisis, the United States experienced a gradual increase in suicide rates at a yearly average of 0.12 per 100,000. Amid the 2008–2010 recession, this rate surged by 0.51 per 100,000, resulting in an estimated excess of 4,750 suicides (Reeves et al., 2012). In countries such as Greece, mainly affected by the financial crisis and austerity policy, there was a 40% increase in suicides between January and May 2011 compared to the previous year (Karanikolos et al., 2013). In this extant literature, there is a significant gap in studying institutional responsibility for the prevention of work-related suicides. To address this gap, in this paper, we explore institutional responsibility in work-related suicides and their prevention.

To address the gap in the literature on institutional responsibility, we draw on Durkheim’s foundational work on suicides. Based on two axes of social regulation and social integration, Durkheim (1897/2010) identifies four types of suicide. If social integration is high, individuals tend to attempt altruistic suicide, ending their lives for the sake of others is a form of self-sacrifice. If their social integration is low, they may attempt egoistic suicide, ending their lives for personal rather than social reasons, such as wishing to end their suffering. On the social regulation axis, if there is a high level of social regulation, suicide may be fatalistic, as the individual sees no other alternative in the social constraints imposed on them, such as bars on their access to a decent life. If there is a low level of social regulation, suicide may be anomic due to the decline of social institutions and measures to prevent suicides. In this paper, we focus on anomic suicide in a country where social regulation is low. Instead of focusing on social regulation at the macro-social level, we set our gaze at the meso-institutional level of regulation and lack of institutional practices, policies and discourses of suicide prevention.

We locate our study in Turkey, a country with declining social regulation and worker protections and an increasing record of suicides at work. According to the Health and Safety Watch (İSİG) (2022), there have been 649 cases of suicide among workers in Turkey over the past 9 years. This context of anomic suicides has its roots in the neoliberal expansion and entrenchment in the country, which stalled and regressed its social protection measures (Yücel and Kabalay, 2023). Turkey has been experiencing a period of decline in social regulations and concomitant institutional practices to prevent suicides. As a result, Turkey provides an interesting national context for studying institutional suicides due to the failure of prevention and institutional responsibilization. We collect 17 suicide notes of prominent anomic institutional suicides from 2017 to 2023 in line with methods employed in previous studies (e.g., Waters, 2017a,b, 2018) and extensive narratives from institutions where the deceased worked and accounts of their friends, family and colleagues. We have included both public and private institutions as settings for institutional suicides in our sample.

We note that other institutions such as family, education, employment, law and other aspects have regimes that can inculcate conditions of poor social regulation that lead to anomic institutional suicides (Clegg et al., 2016). However, we focus on institutions of economy and employment in particular. We identify two alternative mechanisms of institutional suicide: dehumanization leading to lack of recognition by Honneth (2001) and Haslam (2006) misrecognition by Bourdieu (1997). Honneth’s theory of recognition posits that individuals aspire for recognition to retain their dignity and pride at work and in life. Groutsis, et al. (2020) show that a lack of individual recognition may result from the decline of institutions and may push people to migrate in pursuit of better lives. A lack of recognition of human values could lead to dehumanization, according to Haslam (2006). In this paper, we examine suicide as a last individual attempt at highlighting the failure of institutions to deliver such recognition. Bourdieu’s concept of misrecognition is located at the level of the field. When selecting the institution’s microcosm as a field of relations, misrecognition is how institutions denigrate and devalue the knowledge, abilities and skills, i.e., knowledge capital of individuals (Morillas, 2023). Misrecognition is a process through which institutions as a social field can undermine the human potential of individuals. Thus, misrecognition could also cause individuals to feel devalued in institutional settings. While lack of recognition denigrates dignity and humanity, misrecognition devalues human potential and resources.

Our study identifies four different forms for each of the institutional suicide mechanisms: demands for recognition, struggles for recognition, coping with lack of recognition, and ultimate suicide due to the institutional denial of recognition; asserting human potential, undervaluing human potential, negotiating human potential, and misrecognition of human potential. These institutional suicide forms, structured as a process, reflect hopes, aspirations and potentiality of individuals meeting suicidal institutional structures and how this encounter fosters institutional suicides. We also find that suicidal institutions often turn to denial of the conditions mentioned in the suicide note. We turn to the sociology of ignorance (Gross and McGoey, 2015) to explain why suicidal institutions choose to ignore institutional responsibility for suicides and how responsibilization (Vincent et al., 2024) could prevent future suicides. We posit that re-building institutions requires institutional responsibilization shaped by demands in the decedent’s wishes to prevent future institutional suicides. Current practices of individual responsibilization in cases of institutional suicides leave individuals exposed to suicide risk and erase institutional responsibility altogether by reducing suicide to an individual act.

### Theorizing institutional suicide: dehumanization due to lack of recognition, misrecognition, and responsibilization

The “suicide epidemic,” which started in the mid-2000s, found a widespread response in France in companies such as France Telekom, Renault, Thales, IBM, La Poste, HSBC and BNP Paribas (Coupechoux, 2009; Lichfield and Mauviel, 2009; Germain, 2013; Chabrak et al., 2016; Waters, 2017a, 2018). The suicide epidemic happened also in Telstra in Australia (Germain, 2013), Foxconn in China (Chan and Pun, 2010), Huawei, and Samsung in South Korea (AsiaNews.it, 2010), JP Morgan Co Facebook, Google, Bank of America Merrill Lynch, UPS, Activision Blizzard in the US (Davies, 2014, Rodriguez, 2019; Garcia-Roberts and Liao, 2022; Sainato, 2022), Zurich Insurance in Switzerland (Neate, 2013), and Panasonic and Toyota in Japan (The Japan Times, 2019), providing current and live evidence of the seriousness of workplace suicide (Tan and Xia, 2023). Today, the workplace has become the primary site of social suffering where the atrocities of the economic order are felt most intensely and acutely (Kaspar, 2012). The workplace has now been transformed into a site of violence and elimination, where workers are considered by management to be a weak and inefficient factor of production and where there are acts of cruelty designed to devalue their contributions and humanity, eliminating social solidarity (Waters, 2017b). There is a sharp increase in workplace suicides in the context of deteriorating working conditions linked to job insecurity, job intensification, the decline of trade unions and reduced social protection. While workplace suicide is subject to a negationist tendency that denies links between the act of suicide and the workplace, suicides cannot be explained solely in terms of individual causes. Therefore, there are causal links between workplace conditions and suicides (Choi, 2018; Waters and Palmer, 2022). To return responsibility for preventing suicides back to institutions, we frame cases of suicides at work due to declining conditions of work and denigration of workers’ humanity and potentiality as institutional suicides.

Suicide is often defined through dominant religious, moral, social and cultural lenses that tend to stigmatize or valorize the person who commits it. The institutional settings which foster suicidal outcomes are often ignored, leaving institutions with conditions that lead to suicides unaccountable. Suicide is not only an individual act but often a social and institutional phenomenon. Suicide is at the nexus of individual choice to end life that connects with a set of circumstances in social and institutional settings. Although suicide is an individual act, like all human acts, anomic suicides happen in certain social and institutional settings. Institutional suicide is a form of suicide that results from policies, practices and discourses that fail to recognize human values and potential and misrecognize individuals, dehumanizing, denigrating and devaluing them. Similar to allied terms such as institutional sexism (Yih, 2023), institutional racism (UK Parliament, 2021), and institutional homophobia/transphobia (Maughan et al., 2022), institutional suicide refers to institutional policies, practices and discourses that generate suicidal outcomes. Even though individual actors and leaders in an institution may not wish to cause suicides or may wish to prevent suicides, an institution may generate suicides through its policies, practices and discourses.

There are two mechanisms of institutional suicide. One is the mechanism of recognition (Honneth, 2001) that individuals pursue meaning, freedom and dignity at work and in life. Honneth (2000) shows that recognition, that is, approval of the positive qualities of individuals or groups, enables people to be independently satisfied and gain self-esteem. Each individual needs the other to be recognized since they cannot develop their identities without being recognized by others with whom they interact. However, it is difficult for individuals with weakened self-esteem to face negativities alone, and they need support that can make them feel strong enough to cope (Pierson, 2011). Therefore, the recognition mechanism could lead to suicides if institutions fail to recognize the individual needs for dignity, respect, freedom, and equality. Dominant systems within the institutions may fail to recognize individuals’ human qualities, leading to a sense of worthlessness and vulnerability (Bell and Careen, 2011). In this context, dehumanization is a pervasive psychological process where affected individuals are stripped of their intrinsic value and dignity, exacerbating mental health issues and increasing the possibility of work-related suicide. Institutions that fail to recognize individuals’ values, purposes, and dignity contribute to two forms of dehumanization: animalistic and mechanistic. Animalistic dehumanization categorizes individuals as basic or primal, while mechanistic dehumanization renders them akin to emotionless machines (Haslam, 2006; Haslam and Stratemeyer, 2016). Lack of recognition by institutions could lead to two forms of dehumanization.

The other mechanism of institutional suicides is misrecognition. Bourdieu (1997) defines misrecognition as a process through which a social field (institutions in this case) may devalue, undermine, or denigrate the symbolic value of an individual and their human potential. Individuals have potentials such as varied forms of capital (social, economic, cultural and symbolic capital), work-related insights, experiences and aspirations, and drive and agency to reach their full potential. Misrecognition as an institutional mechanism could undermine and devalue the overall endowments of individuals, locking them into paths at work below their levels of skill, knowledge and potential. Dominant groups may often use misrecognition as a strategy to keep subordinate or out-group members away from positions of power and authority and fulfilling paths of career and work. The failure of institutions to deliver recognition through dehumanization and their misrecognition of human potentiality are two distinct mechanisms that lead to institutional suicides.

Institutional policies, practices, and discourses shape collective human activities and routines that have the potential to generate a wide range of positive and negative outcomes, such as improving wellbeing to generating inequalities, despair and suicidal thoughts and actions (Kawachi and Berkman, 2000; Mergen and Ozbilgin, 2021a,b). This paper explores the dark side of institutional policies, practices, and discourses, focusing on suicidal outcomes. We define suicidal institutions through certain policies, practices and discourses that lead to suicides. Our closer examination highlights the nexus of suicidal institutions, unveiling a complex interplay of factors. Individual predispositions intertwine with systemic, institutional influences, escalating the possibility of suicide. Situations of mobbing, violence, and bullying are deeply embedded in such institutional contexts, compounded by intense stress, overwhelming workloads, and inadequate support systems (Lorettu et al., 2020). An institution environment tainted with such incivility amplifies the mental health deterioration of afflicted individuals (Follmer and Follmer, 2021). The impact is not insular but radiates throughout the organization, precipitating a decline in collective morale and productivity (Tan and Xia, 2023). Gabriel (2012) uses the term miasma to account for institutional contexts which create states of darkness. Institutional suicides manifest in suicidal institutions that fail to take responsibility for preventing suicides and inculcate poorly protective and declining work conditions for individuals.

### Locating Turkey in an international context of workplace suicides

The literature primarily focuses on work-related suicides in the USA, France, Japan, the United Kingdom, Australia, India, China and South Korea, addressing them mainly at the individual and institutional levels (e.g., Amagasa et al., 2005; Peek-Asa et al., 2021a; Jang et al., 2022; Waters and Palmer, 2022; Sarpong, 2024). In addition, studies primarily aim to group work-related suicides according to type or profession or to reveal the triggers of work-related suicides, and these studies attract more attention from the fields of psychiatry, psychology, and organizational behavior. Institutional perspectives on suicide are limited, with few exceptions (e.g., Clegg et al., 2016). In the USA, 12.1% of the 84,389 suicides between 2013 and 2017 were work-related. Individuals who attempt work-related suicide, reportedly had financial problems, depression, being evicted, or losing their home. Suicide prevention policies recommend workplaces offer services such as financial planning and psychological support to their employees to prevent work-related suicides (Peek-Asa et al., 2021a). Another study conducted in the USA compares death certificates and death investigations and discovers significant inconsistencies between the two documents. For example, while only 2.1% of suicides among those working in protective services were listed on the death certificate as work-related suicide, the cause of death was cited in 15.2% of death investigations of the same group, indicating that work-related suicides were much higher than documented (Peek-Asa et al., 2021b). In another study of physician suicides in the USA between 2013 and 2018, examined by integrating natural language processing and thematic analysis, five themes were identified in work-related physician suicides: inability to work due to poor physical health, substance use that compromises employment, the interaction between mental health and work-related issues, legal issues leading to work-related stress, and increased financial stress (Kim et al., 2022). Work-related suicides in the United States increased by 22.2% between 1995 and 2010, becoming one of the country’s leading causes of death. Although organizations have responsibilities for their employees’ physical and psychological wellbeing, they do not report institutional accountability (Germain, 2013).

Recent civil society engagements have the potential to change this picture by bringing society’s attention to work-related suicides. USA-based NGOs like the Suicide Prevention Resource Centre and the National Institute of Mental Health argue that there is a comprehensive approach to reducing the number of suicide deaths, and this approach involves multiple stakeholders, including employers. Employers can prevent suicide attempts by providing mental health and wellbeing facilities and considering workplace factors influencing suicide risk (Howard et al., 2022). The connection between mental health, wellbeing, and suicide is undeniably intricate and multifaceted. Although not all individuals grappling with mental health challenges are prone to suicide, a discernible and robust correlation exists between mental health disorders and the risk of suicidal thoughts and behaviors. Even in efforts of suicide prevention charities, there is limited provision for institutional responsibilization for prevention.

The prevalence of work-related suicides has also been escalating across diverse hierarchical levels within organizations in France. Research involving 87 respondents aged 18–65 who had been hospitalized following suicide attempts pinpointed the causal factors to be the quality of relationships with superiors and colleagues and a lack of recognition rather than conditions related to the physical work environment, hours, or contract type (François et al., 2012). Enhancing social relations and recognition within the workplace emerges as a critical preventive strategy. Concurrently, another study highlighted that 5.2% of male and 5.7% of female workers in France experienced suicidal thoughts within a year, associating this with psychosocial factors including diminished meaning and community at work, role conflicts, and job insecurity (Niedhammer et al., 2020).

France was one of the countries hardest hit by the Covid19 pandemic and remained with many cases. Debout and Sanaullah (2022) discussed a survey result carried out on behalf of the Foundation for European Progressive Studies (FEPS) and Foundation Jean Jaures (FJJ) among six European countries, i.e., France, Germany, Spain, Poland, Sweden, and Ireland, with 1,000 people from each country. The survey investigated mental health and suicide during the pandemic and shows that people in France are more likely to act on suicidal thoughts than in the other five countries. Individuals in adolescence and young adulthood, the unemployed, and those engaged in unstable or hazardous working conditions have exhibited an increased propensity for suicide attempts. A study encompassing 1,000 French participants revealed that approximately one-third engaged in suicidal behaviors during the pandemic. Within the subset of respondents experiencing suicidal ideation, 33% attributed it to intense occupational stress, 39% to the experience of burnout, and 37% to enduring moral or sexual harassment within their professional environment. These findings underscore the significant impact of employment status and workplace conditions on mental health outcomes amidst the ongoing health crisis.

Occupational categories associated with excessive and heavy workloads revealed low social support, high psychological demand, intolerance, and long working hours as reasons for work-related suicides, known as *karojisatsu* in Japan (Milner et al., 2018; Waters and Palmer, 2022). Additionally, the Japanese business management system, characterized by lifelong employment, seniority-based pay scales, and loyalty to the employer, triggers work-related suicides as well (Amagasa et al., 2005). In Japan, more than half of the individuals commit suicide within a month of developing a mental disorder, and the suicide rate is higher among administrative and professional or engineering workers. Interpersonal conflict within the workplace serves as the primary catalyst for work-related suicides among individuals who are confronted with issues of chronic overwork and long working hours (Nishimura et al., 2022). An analysis of suicides in South Korea showed a prominent cluster characterized by managers with excessive responsibility, termed as responsibility-burdened type of suicide (Jang et al., 2022).

In the UK, the biggest problem is that suicides are not recorded, investigated, or regulated (Waters and Palmer, 2021). For instance, while even a work accident such as an arm fracture is reported to the relevant institution, such a procedure is not followed for work-related suicides. Unfortunately, the fact that employers are not required to investigate the reasons for an employee’s suicide and to adjust workplace policies following the suicide means that the risk of work-related suicide continues for other employees (Waters and Palmer, 2022). Although work-related suicides are not monitored, mental health problems have been a much-discussed topic over the past decade in the UK, costing the UK economy at least £ 117.9 billion annually (McDaid et al., 2022). NHS (National Health Services) Digital Mental Health Services Monthly Statistics support the above value and highlight the rising demand for mental health services. In May 2023, the number of adults contacted in mental health services was around 1,130,773 (NHS, 2023). While this monthly figure reveals the need for systemic, comprehensive, and sustainable interventions, particularly at workplaces, to reduce or prevent mental health problems causing suicide, CIPD (2021) reports that only 50% of UK-based organizations that participated in the research put employee wellbeing in their corporate agendas. The report’s list of the most common top three causes of stress at work aligns with Waters and Palmers’ case study (2021) regarding work-related suicide factors. Unmanageable workloads, management style and changes in work status are seen as causes of stress and risk of suicide. Although the UK has a national-level strategy to prevent suicide (Garrat et al., 2023), the current numbers address a need to prioritize mental health awareness, early intervention, and support systems to reduce the risk of suicide among those struggling with mental health challenges.

If we contextualize the above to explore workplace suicides, we need to start with the decline of social regulation and social welfare. Social regulation is under considerable backlash internationally. Populist movements that attack institutional mechanisms that provide social safety and security for individuals are on the rise. Suicidal episodes with anomic traits highlight deep social issues. Anomic suicide severity indicates underlying unrest in various cultures (Clegg et al., 2016). Ideologically, neoliberalism provides a fertile ground for the deregulation of social welfare systems, fosters the rise of individualism and corrodes sociality by overplaying the financialization of social life (Valsecchi et al., 2023). Stiglitz (2012) argues that industrialized countries that have taken the neoliberal and capitalist expansion route made their institutions and sectors of work responsible to combat adverse impacts on human rights and equality for their citizens. However, not all industrialized countries have responsibilized their industries and institutions for suicide prevention. For example, situated within a Scottish framework, Holligan and McLean (2019) posit that the tenets of neoliberalism deepen societal disparities. This exacerbation precipitates heightened anomie and profound alienation, with the gravest consequences manifesting predominantly within the marginalized sectors of the population.

Many countries of the Global South are even less attuned to responsibilizing other institutional actors for their citizens’ human rights, health and safety and dignity concerns. Turkey provides a worrying example as a country where neoliberalism is practiced without mechanisms of responsibilization (Kusku et al., 2021, 2022). Consequently, the Turkish experience of neoliberalism has exposed workers to excesses of insecurity, indignity, precarity and inequality without an institutional mechanism of safety (Camgoz et al., 2023; Erbil and Özbilgin, 2023). Neoliberal policies have disproportionately impoverished minority populations and, therefore, must be considered a possible factor influencing suicide rates (Zeira, 2022). Turkey’s economic liberalization project dates back to the 1960s, and the neoliberalization effort goes back to the 1980s (Erbil and Özbilgin, 2023). While the neoliberal project denigrated the meaning and purpose of all public sector organizations, shifting them toward private sector logic, it has also hit social and workplace protections in Turkey that provided safety nets to individuals, exposing them to precarity, indignity at work and widening income gaps. Within the Turkish milieu, long-established merit-based frameworks have traditionally advocated for competencies and demonstrable accomplishments, ensuring the rightful acknowledgement of individual education, talents, skills and abilities. However, emerging trends suggest a concerning deviation from these foundational standards. Replacing these traditional norms, *biat* (fealty in Turkish) has emerged as a distinct paradigm in contemporary institutional recognition systems, distinguished by its ingratiatory tendencies emphasizing political allegiance (Özbilgin et al., 2019; Özbilgin and Yalkin, 2019). While marginalizing genuine expertise, this shift compromises institutional recognition of merit and elicits disenfranchisement among those whose achievements are not recognized. As a result, talented individuals face misrecognition and resultant effects of heightened psychological distress, further potential vulnerabilities to severe mental health challenges, including suicidal tendencies. The repercussions of this systemic deviation are gravely evident; for instance, over a span of a decade in Turkey, approximately 300 teachers who, despite their qualifications, remained unappointed to official positions have attempted suicide, resulting in death (Kırcı, 2022). While 431 health workers attempted suicide between 2015 and 2017 (Çiftçi, 2021), the number of police officers who attempted suicide between 2016 and 2022 was 347, 17.4 per 100,000, much higher than the population average (4.1) (Toprak, 2022). Such circumstances highlight the deep psychological turmoil induced by these institutional contexts and the absence of their acknowledgement.

The shame and embarrassment embedded in Turkish societal values influence the challenges faced by individuals grappling with mental health issues, unemployment, or political non-conformity (Satici and Deniz, 2020; Daǧ and Alkar, 2022). The societal expectations placed on individuals to adhere to certain norms and standards also create significant pressure, contributing to the dread of judgment and stigma associated with these problems (Muftuler-Bac and Muftuler, 2021). Turkish cultural context exacerbates feelings of isolation and despair among those who feel they do not meet these expectations. In this cultural context, reluctance to admit to mental health struggles or socio-economic difficulties often stems from a perceived notion of weakness or failure directly influenced by the weight of these societal expectations. This pervasive silence around such issues reinforces a societal norm where mental wellbeing and personal struggles are rarely discussed openly. As a result, Turkish cultural tendency contributes notably to the underreporting of mental health concerns and suicides in Turkey, with individuals and families often choosing silence over seeking help or acknowledging such issues, thereby perpetuating a cycle of distress.

Since the 1980s, Turkey has been going through an untamed form of neoliberalism (Stiglitz, 2012), which manifests as the emergence of a set of toxic institutional norms and behaviors (Kusku et al., 2021, 2022; Mergen and Ozbilgin, 2021a,b). Leadership culture in Turkey involves a level of paternalism, i.e., parental care and concern for the others. Paternity affinity with authority and hierarchy (Aycan et al., 2013) took an adverse turn through neoliberal turn with market rationales overpowering serv and compassionate rationales. Özbilgin and Yalkin (2019) revealed that *biat* (i.e., fealty in Turkish for subservience to authority) emerged as a toxic cultural demand for career success in Turkish organizations. Similarly, in the public sector, Özbilgin et al. (2019) reported that ingration and sucking up behaviors are normalized at work. Overall, in the period of neoliberal expansion, Camgoz et al. (2023) and Erbil and Özbilgin (2023) note that worker silence in the face of declining working conditions is endorsed, normalized and brutally enforced. The institutional and cultural transformation of working lives contribute to further dehumanization and misrecognition for workers from marginalized backgrounds.

Misrecognition in Turkey has sometimes left various groups feeling marginalized, potentially contributing to feelings of alienation and heightened mental health concerns (Baykut et al., 2022; Palalar et al., 2023). Turkey has many cases of recorded suicides. However, research (Coskun et al., 2012; Yüzbaşıoğlu and Işık, 2019) also identifies that there could be extensive underreporting of suicides by individuals from vulnerable communities. The challenges faced by women, ethnic minorities, the LGBT+ community, political opponents of the ruling elite, and religious minorities like the Alevis, individuals with disabilities not only limit their societal participation but might also impact their emotional wellbeing (Özbilgin and Erbil, 2021; Kamasak and Palalar, 2024). For instance, societal prejudices encountered by LGBT+ individuals could lead to increased feelings of despair, escalating vulnerabilities toward suicidal thoughts (Yılmaz et al., 2022; Özbilgin et al., 2023). Transgender individuals face discrimination and violence, with constrained job opportunities, often leading to amplified mental health challenges, including increased suicide rates (Yüksel et al., 2017; Başakçıoğlu, 2020). A lack of proper representation and acceptance may amplify feelings of isolation, a known risk factor for suicidal tendencies.

## Methods

Drawing on a multi-case study methodology, we selected 17 prominent cases of institutional suicides resulting in deaths in Turkey in the last 6 years. As this is a qualitative study based on thematic analyses of suicide notes, the study seeks to identify common patterns in the sample. To improve the distributive elements of the sample, we made sure to include suicide notes of decedents from different genders, ages, social and economic class backgrounds, sectors of work, and professions. We have gathered evidence on suicide notes from decedents, narratives from their families, friends and colleagues and responses and public announcements from workplaces. The availability of suicide notes of the decedents and statements from their friends or families in media significantly influenced our case selection. An additional criterion we considered was our ability to access responses from the institutions associated with the suicide incidents. To maintain confidentiality, respect the sensitivity of the information, and protect families and friends of suicide decedents, we anonymized the identities of both the decedents and their institutions, in accordance with the publication policy and ethical principles of the journal. To preserve the anonymity of the decedents, we used gender-neutral pseudonyms and adopted a gender-neutral language. We included suicide decedents from public and private sectors and diverse socio-demographic profiles. Of the suicide decedents, seven were employed in the security sector, while three worked in education. The remaining individuals were affiliated with various industries, including logistics, culture, health, religious services, aviation, and banking. These institutional suicides occurred between the years 2017 and 2023.

The multi-case study method is particularly helpful in identifying social mechanisms that form the antecedents of institutional suicides, as we were able to collect data from the decedent’s suicide notes as well as institutional responses. As our study explores the interplay between personal experiences and institutional practices, the multi-case study method allows us to examine micro-individual concerns in the context of meso-institutional processes and practices (Stake, 2013).

Once evidence was collated for each case, we used content analysis techniques to analyze the data. Content analysis enabled us to systematically analyze and interpret the contextual data extracted from suicide notes and institutional responses (Stemler, 2000). We employed the coding strategy (Vollstedt and Rezat, 2019) to explore and cut data across two mechanisms: dehumanization due to lack of recognition and misrecognition as antecedents of institutional suicides. We applied coding schemes to categorize data and unveil emergent themes, establishing connections to institutional practices and individual experiences. This analytical approach offers valuable insights into the social and organizational dimensions underlying the incidences of suicide, supporting a more nuanced and integrative comprehension of the phenomenon. Table 1 outlines our data structure.

**Table 1:** Codes, subthemes, and themes.

Codes	Subthemes	Themes
“Hope,” “expectation,” “virtue,” “empathy,” “honour,” “success”	Denial of recognition	Dehumanisation
“Incivility,” “disappointment,” “desperation,” “repelling,” “dismissal”	Struggle for recognition	
“Alienation,” “frustration,” “denigration,” “burnout,” “lack of purpose”	Coping with lack of recognition	
“Exhaustion,” “stagnation,” “loss of enjoyment,” “farewell,” “desire for escape”	Ultimate suicide	
“Injustice,” “loss of reputation,” “violations,” “ethical concerns”	Asserting human potential	Misrecognition
“Insulting,” “stress,” “worthlessness,” “disillusionment”	Undervaluing human potential	
“Dissatisfaction,” “imposition,” “enforcement,” “coercion”	Negotiating human potential	
“Accusation,” “avoidance of responsibility,” “insinuation,” “evasion”	Blaming	Institutional responses
“Manipulation,” “dishonesty,” “negation,” “obfuscation”	Denial of immediate responsibility	
“Institutional interests,” “institutional confidence,” “institutional image”	Prioritization of protecting reputation	
“Transparency,” accountability,” “scrutiny,” “monitoring”	Promise to investigate	
“Absence of awareness,” “absence of advocacy,” “apathy,” “insensitivity of warnings”	Lack of commitment to suicide prevention and institutional change	
“Belonging,” “forgiveness,” “remission,” “atonement”	Individual responsibilization for prevention	Last wishes as co-design
“Rewarding,” “voice,” “combatting,” “compensation,” “compliance,” “leadership”	Institutional responsibilization for prevention	

### Findings

We identified four themes. Two of the themes refer to the institutional suicide mechanisms drawing on concepts of dehumanization due to lack of recognition (Honneth, 2001; Haslam, 2006) and misrecognition (Bourdieu, 1997). Individuals have human values and desires to have dignity, meaning, purpose, and justice. They pursue recognition for their values. Denial of recognition of their values could lead to dehumanization and suicidal tendencies for individuals. Individuals also have varied resources, such as social, economic, cultural, and symbolic capital, constituting their human potential. Institutions are uniquely positioned to recognize human values and evaluate human potential. Suicidal institutions in our study have two distinct mechanisms: lack of recognition of human values leading to dehumanization and misrecognition of human potentiality. The third theme is the institutional denial of responsibilization. Drawing on the sociology of ignorance (Gross and McGoey, 2015), we framed denial of responsibilization as an act of ignoring suicides. The fourth theme in this study is “the last wish as co-design” which we describe and present as a future policy for institutional responsibilization (Vincent et al., 2024) based on what the deceased expressed for preventing institutional suicides.

### Mechanism 1: dehumanization as the denial of recognition of human values

Individual aspirations for meaning, purpose and realizing a life with dignity, and human values met institutional structures at work (Laaser and Bolton, 2022). Workplaces that value and recognize their workers’ inspirations, aspirations, values, and desires tend to support employee wellbeing and welfare. On the dark side of worker-workplace relationships are workplaces which deny recognition of human aspirations, desires and needs for dignity. Honneth (2001) refers to recognition as a central drive for individuals to thrive at work and in life. In all suicide notes we studied, we identified themes that show the process of demands for recognition, struggles for recognition, coping with lack of recognition, and ultimate suicide due to the institutional denial of recognition. We outline below these forms of lack of recognition in their sequential order, as expressed by suicide decedents and their relations.

#### Demands for recognition

Suicide notes that we examined have implicit or explicit statements of the demand for recognition of the suicide decedent’s values, aspirations and needs. In suicide notes, demands for recognition sometimes emerged as statements of their fit with the institution. How they enjoyed certain parts of their roles at work, and how they flourished, Thus institutional suicide does not only have a dark side. Institutions provide more than suicidal conditions often. It is the partial nature of recognition that makes often invisible the role of institutions in generating suicidal outcomes. One suicide decedent, Evren, explained how they enjoyed part of their work, where they found meaning, purpose, friendship, professional fulfilment and even love at work.

I started my professional life [institution]. I built positive relationships with everyone …. I can say that it was a pretty intense and successful period. Frankly, we could call it a career start that aligned perfectly with all the principles we advocate for in the organisation. A few years into my career, I wanted to change teams and work on a larger project. […] These years, filled with accomplishments, brought me to what I believe is the earliest managerial position in the institution’s history. I’m sharing this to emphasise our positive journey, a point that would fulfil anyone’s dreams. Additionally, in this institution, I met one of the most valuable people in my life, my cherished spouse. (Evren).

Another suicide decedent Ekin explained how attempted they were to professional virtues and how they declined to receive an honorary title they were offered because they felt they did not deserve it. Their story signaled how they were committed members of this profession and wished to be remembered as such. However, suicide decedents also reported the human qualities that made them susceptible to suicide in their institutional context. For instance, Fikret mentioned in their note how their soft nature made them more susceptible in the context of the institution. Yet, they tried to retain a certain level of integrity with human values. Demands for recognition of human values that the suicide decedents had are stated either explicitly or implicitly as above. The next theme delves into how their demand for recognition turns into struggles for recognition when their demands meet their institutions’ policies, practices and discourses.

#### The struggle for recognition

Institutions’ denial of individuals’ dignity and pride does not always prompt individuals’ reassessment of expectations. Denial, mistreatment, and challenging communication in the workplace culture intensify the individuals’ quest and struggles for recognition (Burke-Smalley et al., 2022). Constrained by a conflict between self and institutional values, individuals encounter significant obstacles when attempting to counterattack institutional systems that undermine their values. In turn, the struggle for recognition may metamorphose into a catalyst for dehumanization, leading to suicide. One decedent mentioned losing their emotions while struggling to make the institution recognize their expectations. Their experience of the progressive loss of emotions in their effort to compel the institution to acknowledge their expectations illuminated this tragic outcome.

Dehumanization manifested as acts of incivility, violence and aggression, as the suicide decedents noted. Ülkü explained how they could not cope with fear and the experience of being scolded at work. Dehumanization is also manifested as diminishing individuals’ self-worth, sense of identity, and public denigration and devaluation. Evren noted their experiences of losing a sense of control through this process. Another suicide decedent struggled with fitting into the religious establishment, where modern life choices and attire were considered inappropriate. Common to most cases of dehumanization is the top-down imposition of value and scant room for negotiation for the individual (Rader, 2008). Once the institution makes a decision, there is hardly any mechanism of appeal or redress for the individual. Dehumanization is almost entrenched in the institutional mechanism. Struggles of recognition, for those decedents who attempted this, failed. They were made to feel powerless to assert their human values.

#### Coping with lack of recognition

When faced with the dehumanizing treatment of the institution, decedents reported resigning to cope with the results of the dehumanizing experience. Coping, unlike thriving or enjoying work, is an aversive state. Individuals cope when the conditions are less than ideal. Coping often leads to feelings of frustration, hopelessness, alienation, and loss of meaning (Dewe et al., 2010). Eser wrote how they felt trapped in a life which was not their choosing. Some suicide decedents wrote how dehumanization was worse than other forms of oppression and disadvantage with material conditions. The clash with institutional values denigrated the honor and dignity of the suicide decedents led to feelings of hopelessness.

I can live without food or water, but I cannot live with the humiliation made to my honour, dignity, and cause. […] In our organisation, certain things are being covered up due to people’s egos and worldly concerns. (Eser).

Dehumanization and lack of recognition of human values lead to negative feelings, the most prominent of which appears to be hopelessness due to the absence of mechanisms of redress and measures to combat dehumanization. The next phase is the ultimate end, the suicide.

#### Ending own life as the ultimate response to dehumanization

Ending one’s own life is not a light decision. The suicide notes show the complexity and multifaceted nature of this decision. Some suicide decedents noted suicide as their own failure to live with the dehumanization espoused by the institution. Göksel wrote about how their experience of dehumanization at work drove their joys away and rendered their life project a failure.

I cannot enjoy life. I’ve felt this way several times before, but this feels different. I’ve lost hope for the future. I do not think I’ll ever enjoy life in the future. I constantly strive to live, grappling with challenges. This is now wearing me down. Being happy seems so meaningless. I’m exhausted. No matter what I achieve or possess, I feel as if I have nothing. In other words, the things I own do not bring me happiness. […] It’s been a long time since I learned any information that would truly excite my brain. […] Life has become so tedious that I do not even have the patience to live long enough to learn them. (Göksel).

Institutional practices of dehumanization and lack of recognition dig deep into dark states of mind for the suicide decedents, often making it impossible to see any glimmer of hope. For some decedents, their demands for recognition of their aspirations met with dehumanizing norms of the institution that denied such possibilities to them. Institutional limitations on freedom of thought, belief and expression emerge as a common theme that dehumanized the decedents and pushed them to a feeling of helplessness and entrapment. Dehumanization leads to individuals’ alienation from their own identities and values and may foster conformity to environments that neglect their inherent worth. Some suicide notes indicate that the decision came after profound exhaustion stemming from this pervasive alienation. After various attempts to gain recognition for their struggle, Evren expressed how they were exhausted and could not continue any longer after a long struggle with the denigrating terms and conditions of work.

Religiosity may lead individuals to interpret challenges as tests framed within their specific value systems (Durak, 2011). Some view confronting hardships as a route to personal growth, while others navigate them with a sense of resignation, grounded in the belief that there will be a reciprocal relief for their struggles. Suicide notes revealed that many wrestled with their adversities armed with a hope rooted in religious conviction. However, such faith did not prove potent enough to restrain them from the act of suicide. Religiosity may exacerbate dehumanization when it obstructs individuals’ ability to question the erosion of their values, leading to an increased sense of alienation from themselves and their institutions. The note from Hazar exemplified how they struggled to reconcile their personal and professional values and dignity with the conditions offered. The normalization of practices that are unethical, illegal, or incongruent with human dignity resulted in the dehumanization of individuals. The absence of voice mechanisms, lack of protective measures, and absence of legal regulations contribute to the normalization of rights violations (Camgoz et al., 2023). In Olcay’s note, they detail how silencing mechanisms within an institution that dismissed their rights ended with them taking their own life. The belief in justice systems supports individuals in defending and upholding their values and dignity. However, when institutions fail to recognize individual values, the ensuing discouragement and the erosion of faith in the justice system may drive individuals to make radical life decisions. This lack of recognition and protection possibly undermines their enthusiasm to pursue their life plans. Evren’s suicide note concluded with sentences that unveil an erosion of trust in the justice system.

### Mechanism 2: misrecognition

All institutional suicide cases in our study reveal ways that institutions misrecognized the cultural, social, economic and symbolic worth and potential of the decedent by subjecting their human potential to misrecognition. All humans bring their human potential to work. This includes their varied forms of capital such as different ways of knowing, education, training, experience, social and professional networks, and financial and symbolic resources. However, our analysis shows that suicidal institutions misrecognize individual knowledge resources. In suicide notes and other documents, it is possible to see that individuals would like their knowledge, skills, and abilities to be valorized and fit for purpose. Suicide notes suggest that there is a process of negotiation and ultimate failure and suicide.

#### Asserting human potential

All suicide decedents reported asserting their human potential and demanding the just and fair recognition of what they bring to work. Suicide notes had sections that elaborated on the decedent’s fit with the work role and profession. What surprised most decedents was that they were often the only people who admired what they brought to work. Ufuk explained how their qualities made them ideal candidates for the post they were holding beyond life’s necessities to work and have an income:

First of all, I’d like to tell you about myself. I am a child of a highly educated and successful family. I was raised in a peaceful environment of what can truly be called a model nuclear family. I completed my entire educational journey full of achievements and honours on scholarships. There’s no need for modesty - I can say that by Turkish standards, I am in what we might call the “cream of the crop” category in every sense. I’d also like to mention that I have a reality where I am, perhaps, financially comfortable, without any worries or troubles. (Ufuk).

#### Undervaluing human potential

When an individual interacts with an institution, they may be over-admired for what they bring to the table, valued for what they offer, or undervalued in terms of their potential at work. Those unjustly favored individuals could also suffer from a mismatch with the high status they are offered. Those who are devalued may struggle to reach their full potential. In the case of our decedents, misrecognition and devaluation of their human potential took several forms. Some experienced this misrecognition as mobbing, harassment, denigration and even violence, depending on the sectoral norms of engagement. When individuals feel treated above or below their potential, this can significantly affect their wellbeing, inducing stress or tedium. One suicide decedent, Hazar, told how their health and wellbeing suffered with the denigration of their passion and commitment to the profession. Misrecognition of what they brought led to alienation, costing health and wellbeing losses. Undermining, devaluing and denigrating what an individual brings to work may flourish in institutional settings where managerial control and power is left unchecked. The family of Bilge explained how the lack of accountability of senior management led to the bullying and mobbing that the decedent experienced to remain uncontested.

#### Negotiating human potential

We note that decedents attempted to negotiate the misrecognition that they experienced and demanded or expected their institutions to recognize their true worth. However, almost all attempts at negotiations of worth failed. Misrecognition appears to be a stronger mechanism that the suicide decedents could combat through their individual or other collective means. Ufuk explained how they felt a deep sense of despair after attempting to combat how the institution undermined them. The suicide decedents also explained how the institutional policies, practices and discourses failed when they and others attempted redress for misrecognition. Ufuk wrote that their efforts to alert the institution to its detrimental processes failed and that they remained without a voice. Misrecognition did not only happen through devaluation of work but with expectation and imposition of overtime and inhuman work conditions that made it challenging or impossible for the suicide decedents to cope. The family of Ayhan’s wrote about their struggles with harsh conditions of work at their workplace, explaining how Ayhan attempted to negotiate their human potential at work.

#### Misrecognition of human potential ultimately leads to suicide

When the suicide decedents find out that their attempted negotiation with the institution failed, they lose hope. Ziya explained how they experienced professional undermining that led to their suicide. When negotiation fails, suicide becomes a viable option for the decedent. Another decedent explained their ultimate choice to end their own life as a result of the failure of the negotiation and long-term efforts to change their poor conditions of work. Worthlessness was a common feeling induced by long exposure to misrecognition among decedents.

### Institutional responses

Institutional responses to suicide notes and allegations within them contained some interesting aspects. First, some notes involved blaming the suicide decedent and their friends and family. Second, all letters involved elements of denial of immediate responsibility for causing suicides. Third, some of the notes offered possibilities of investigation. Lastly, there was a lack of commitment to institutional change to combat future suicides.

#### Blaming the suicide decedent and their network

Institutions unattuned to recognizing human potential and values perpetuate a climate of anomie, which is sustained even in the aftermath of institutional suicides. The forces that drive individuals toward such actions retain their potency, highlighting the persistent ignorance of institutions even post-suicidal events. Institutional ignorance emerges as a potent mechanism employed to ignore or suppress the recognition demands for human potential. The lack of adequate legislation, the absence of mechanisms for accountability, and the deficit in societal pressure for transparency collectively contribute to the perpetuation of hostile institutional responses. These responses may escalate beyond evading responsibility to actively placing blame on suicide decedents. Institutions may mute calls for accountability by shifting the blame onto suicide decedents, accusing them of having weak disposition, neglecting duties, partaking in illicit or unethical actions, or engaging in defamation. This strategic blame allocation served as a form to deflect scrutiny from the suicidal institutions’ own responsibilities. For example, following İlkay’s suicide, explanations by their attorney underscored the deployment of strategic blaming by the institution as a means to avoid its responsibilities.

#### Denial of immediate responsibility

Media attention and societal focus on the institution may prompt them to respond to suicide incidents. We observed that institutions blaming suicide decedents often respond after receiving signals of potential economic and social backlash from their stakeholders. However, these responses typically involved the denial of responsibilities. Some institutions asserted their innocence by claiming that suicide decedents had not raised any explicit demands or complaints regarding their policies or management styles. Some also engaged in an inquiry into various facets of individuals’ lives as a means of cross-checking the circumstances surrounding their suicides. The leader of a trade union made a statement following Tansu’s suicide, illustrating one such example.

We may not know the exact moment people go through because we cannot experience it ourselves. But one thing we do know is that they did not have any debts weighing on their mind, no troubled relationships, no issues with alcohol, and no family problems. (Trade union leader).

Another approach institutions may employ in their denial of responsibility following suicide incidents is manipulation. Instead of providing explanations for their inadequacies in addressing the conditions that led individuals to suicide, institutions may choose to highlight what they have done for individuals. The response from the institution regarding Hazar’s suicide is an example for institutional manipulation, which suggests measures are taken to check the wellbeing of the decedent. Following suicide incidents, institutions may opt to explicitly deny their wrongdoing rather than resorting to manipulation. However, these institutional responses often attempt to distantly convey that their actions did not lead to any wrongdoing. One of the suicide decedents’ institutiton’s response is one such example of these approaches:

It has been determined through investigations that the tragic incident is not related to mobbing, as claimed in the newspaper and some social media platforms. The assertion […] is not true. (Institutional response).

#### Protecting institutional reputation as a priority

Institutions may exploit their privileged positions within their social and economic settings to silence individual objections against their institutional responsibility (Sims and Brinkmann, 2003). Re-building of institutions requires improving and changing themselves, as well as the interests of managers within the institution in the current status quo, to keep these silencing mechanisms in action. Institutional responses following suicide incidents may exhibit these silencing mechanisms. An example of this occurs when institutional leaders, rather than investigating the factors that contributed to the suicide, issue statements regarding how suicide reports harm their reputation. We observed such a silencing mechanism in a manager’s statement from Bilge’s institution. Institutional responses often alluded to the faith, expressing deep condolences and possibilities of investigation through rather drawn-out and often inconclusive legal procedures.

#### Promise to investigate

In the wake of suicide incidents, some institutions have outspokenly committed to probing the connection between such devastating events and their internal organizational and procedural frameworks, prompted by public and stakeholder reactions. However, we discerned a significant deviation from the fundamental tenets of transparency and accountability. Institutional responses indicated that the proposed investigative endeavors lack clear articulation and a robust framework. Our analysis revealed a concerning trend where certain institutions manipulate investigatory commitments. They asserted that the tragedy arises not from institutional failings but from the decedents’ inability to report the institutional wrongdoings they encountered, thereby deflecting scrutiny away from the institutions’ deficiencies. Following media reports on Evren’s suicide, their institution issued a response embodying this manipulation. The institutional statement deflected responsibility and actively sought to avoid scrutiny. This evasion illuminated a troubling trend where institutions adeptly maneuver to obscure their accountability. The show of care and compassion and virtue signaling after a suicide and incriminating suicide notes are part and parcel of institutional mechanisms to overtly express a commitment to accountability while tacitly avoiding such responsibility. Considering the institutional declarations as evidence, there were a few institutions that explicitly accepted their responsibilities about suicide incidents. Following the statement of Ekin’s family, the institution responded with an acceptance of its involvement:

We consider their statement to be the main point. A person who has reached the point of taking their own life has no reason to make false accusations. There are also witnesses mentioned in the letter. Therefore, it seems that the mobbing allegation is true. The sole cause of this suicide is mobbing and insults. Those insulting words are what drove that child to suicide. (Ekin’s family).

#### Lack of commitment to suicide prevention and institutional change

The audit of institutional practices by institutions after the substantiation of their unlawful or unethical behaviors is indicative of a partially constructive administrative reaction. However, when an institution refuses to acknowledge its misconduct, even when confirmed by legal authorities, and neglects to introduce necessary reforms to enhance individual wellbeing, it may illustrate an institutional ignorance that may lead to a propensity for suicide. In examining how institutions respond to suicide incidents, we observed a pronounced tendency to avoid confrontation and shirk responsibility. We noted that these institutions often chose silence over addressing the institutional wrongdoings explicitly implied in suicide notes. Furthermore, we found that institutional responses lacked any acknowledgement or indication of awareness concerning the urgent need for comprehensive institutional re-building and a commitment to suicide prevention. The case of Olgun’s suicide is illuminative; the subsequent statement by their associated union conspicuously demonstrated the institutions’ continued ignorance and the perpetuation of ignoring the imperative of introspection and systemic reform in the aftermath of suicides. The institution responded expressing their condolences and recognition of the injustice faced without any recourse to re-building the institution to prevent institutional suicides.

### Last wishes as co-design

Based on suicide notes, narratives of relations and institutional responses we identified several last wishes of the suicide decedents, the institutions and relations to formulate a co-design for institutions that can prevent institutional suicides. There were two central themes that emerged in the last wishes for preventing future suicides. First was individual and social responsibilization. Suicide decedents indicated individual and social responsibilization that could have improved the situation for them, preventing their suicide attempt. Second, the suicide notes and evidence from other parties suggested that institutional change and responsibilization is required to prevent future suicides. All but one participant referred to these two levels of responsibility in their suicide notes. Inspired by religious teachings of hope-based faith one decedent demanded eternal justice rather than social and legal redress. Even in Sonay’s suicide note, there were demands from the survivors to take responsibility for each other.

No one is responsible for my death, I have no complaints against anyone, everyone will be punished for their actions in the afterlife. First - May God, then my family, friends and students forgive me. I forgive everyone, please forgive me, too. My wish from my country and relatives is that you please take care of my [family]. (Sonay).

However, including the above and other notes suggested two types of responsibilization for preventing institutional suicides. These involved individual responsibilization and institutional responsibilization.

#### Individual responsibilization for prevention

Last wishes in suicide notes are powerful indicators of what could happen once the person dies. Suicide notes contained different forms of responsibilization for individuals at work, acquaintances, friends and family. One decedent appealed the survivors to stand tall against injustice and oppression:

Please forgive me, and thank you for your understanding. Please continue to support and be a beacon of hope for those still alive. In my final farewell, I bid you adieu, having lost all my emotions. I hope that one day, this world becomes a better place. I hope you prevent the people in your life from abusing their power. The way to do this is to stand tall without fear and not to be enslaved. (Evren).

One suicide decedent identified tyranny and despotic individuals as the cause of their suicide. The co-design idea from their last wish was that they did not want anyone to be in pain. Their last wish indicates that the institution did not have mechanisms to stop insults, bullying and harassment. Dealing with these anti-social behaviors could have served as preventive measures. Another decedent identified mobbing as a reason for their suicide. Anti-mobbing policies are a take away lesson as a preventive measure from their letter. The farewell letter contained a sentiment that they failed as an individual to redress mobbing. The letter offers individual responsibilization in the form of survivors supporting and consoling each other and forgiving the decedent. Demanding forgiveness was a common form of individual responsibilization as many decedents were concerned about the pain and suffering their suicide decision would inflict on the survivors. Suggesting that the solution is not individual responsibilization but could be an institutional change of policies and practices, which we continue to explore in the next section.

#### Institutional responsibilization for prevention

Suicide notes alluded to what was wrong with institutional policies, practices and discourses that led to their suicide. Exploring these letters, we identified co-design ideas to prevent institutional suicides. One decedent identified the unfairly levied financial reward as the only means to motivate staff as problematic. They noted that this leads to poor quality of life for the colleagues. For co-design idea we deduce that quality of life interventions may serve as a suicide prevention measure. One decedent explained how the socio-political absence of freedom of speech and worker protections creates desperate conditions for workers. Co-design idea from their letter is that effective prevention of institutional suicides also requires progressive legal and social normative changes around the institution to force the institution to consider suicide prevention a relevant and priority concern. One decedent explained how dehumanization through denigration of their professional dignity and professional pride led to their suicide. The co-design idea is to have interventions to return dignity and pride at work, considering these two as significant human values, the protection of which could prevent institutional suicides. Another decedent noted several individual expectations from friends and family and requested the doctors to improve work conditions for doctors. Co-design idea from this note is the impact that improved conditions of work could help prevent institutional suicides. After this particular suicide, the Turkish Medical Association appealed to national regulators to improve conditions of work for doctors and medical staff in hospitals to return their professional dignity and allow them to realize the full potential of their work with health, safety and security, working under better conditions.

It deeply wounds all of us to lose a medical doctor who had chosen to be a beacon of hope for people, especially during a time when the world expected to look at life with confidence. We offer our condolences to their [the decedent’s] family, loved ones, colleagues, and healthcare community. In our profession, where we take responsibility for human life and carry out our work with honour, there are intense and exhausting working conditions, long working hours, the wear and tear brought about by emotional and physical burdens while practising our profession, the risk of facing violence, our devalued labour and professional identity, and the insecurity-induced by lack of prospects. (Turkish Medical Association).

In the same way, the workers’ union suggested that institutional changes to combat mobbing moderation of long and intense hours of work could prevent institutional suicides. One of the institutions considered the suicide notes with grave concern, unlike others that denied the evidence. Instead of denial, the institution suggests that an investigation is necessary into the conditions that surround the case. The response is pertinent as it suggests the need for institutional responsibilization. In many of the other cases, individual decedents are blamed for their socio-psychological state, with marked failure to recognize institutional responsibilization. Co-design ideas from this case and others above suggest that institutions could do much to improve their policies, practices and discourses to improve their processes of respecting human values and recognizing human potential.

## Discussion

With our study of 17 cases of institutional suicides, institutional responses to these suicides and narratives of family, friends and others on these suicides, we examined the mechanisms of dehumanization and misrecognition that lead to suicides in the anomic context of Turkey. Within each mechanism we identified four forms of each mechanism and presented them in chronological order. The forms of each institutional suicide mechanism involved demanding recognition and respect, meeting institutional resistance in the forms of denigration and misrecognition, negotiating the terms with the institution and the failure of negotiation leading to suicide. We also explored institutional responses to suicides ranging from denial of institutional responsibility to promise of investigation and institutional change. Our analysis of institutional responses revealed that institutional ignorance is a pervasive element, characterized by a systemic failure to adequately address, comprehend, or even acknowledge the intense psychological and emotional distress experienced by individuals. This neglect further amplifies the anomic conditions and obstructs authentic initiatives for alleviation and prevention. Drawing on the last wishes stated in suicide notes and narrative responses to suicides, we identified co-design ideas for the prevention of suicides including responsibilization of individuals to prevent future suicides and deeper level of institutional responsibilization and accountability for preventing institutional suicides.

Our analysis shows that for each mechanism of recognition, the pursuit of meaning and validation in institutions meet resistance in suicidal institutions. As individuals are often poorly resourced and largely disempowered to challenge failing institutional processes, individual recognition efforts lead to institutional suicides. We call this a bottom-up process, where individuals push for solutions, but individual responsibility is set to fail due to their weaker position in their institutions. The second mechanism is misrecognition, a top-down process of institutional actors misrecognizing the qualities of individuals, causing them to feel and experience devaluation of their capital endowments such as knowledge and experience. Misrecognition refers to the nuanced and symbolic process in which an individual’s or a group’s identity, contributions, and intrinsic value are systematically undervalued within the overarching collective or societal framework (Erbil and Özbilgin, 2024). Misrecognition weakens individual power at work and presents a mechanism of institutional suicide. Similar in both mechanisms is the way the individuals are disempowered in human values and potentiality in their engagement with their suicidal institutions.

Whose responsibility is it to prevent institutional suicides? The mainstream literature on suicides continues to treat suicides as individual acts, with limited recognition of social and institutional responsibilization for the prevention of institutional suicides (Sarigül et al., 2023). Therefore, many measures target individual psycho-social measures to prevent institutional suicides. Our examination shows that suicide decedents identify institutional reasons such as the absence of measures to combat misrecognition and lack of recognition as central causes of them taking their own lives. If we continue to frame workplaces related suicides as autonomous individual acts, we miss the opportunity to recognize institutional and social anomie as a significant antecedent for suicides. We explored suicides in the context of a country with ceremonial human rights laws, weak employee health and safety regulations, absent suicide prevention measures and unsupportive workplace practices. Recognizing anomie as a reason for suicide helps us recognize the accountability of social, institutional and national regulation in causing and preventing institutional suicides. After most suicides, institutions’ denial of responsibility appears to be the norm. Responsibilization of individuals for suicide leaves a vacuum for more effective and systemic changes that could come with institutional responsibility for the prevention of suicides and accountability for addressing institutional and systemic causes.

Our focus on co-design ideas reveals possibilities of recognizing and respecting human values and promoting and recognizing human potential in institutional policies, practices, and discourses that may serve to prevent institutional suicides. Such design, if captured not only by institutions but also as part of social culture and national normative structures, may transform suicidal institutions and prevent institutional suicides (Özbilgin, 2024). There is a significant role for national law makers in building accountability structures in recognition of the need to prevent institutional suicides. Without the coercive power of the law, the Turkish case suggests that institutional responsibilization does not happen in isolation. Normative pressure from law, social and cultural forces and institutional leaders is important for institutional responsibilization (Wicks, 2023). Developing advanced strategies to mitigate institutional suicides fosters conducive policies, practices and discourses that both recognize the need for human dignity and freedom from misrecognition is imperative. A comprehensive elucidation of the multifaceted dynamics intrinsic to institutional suicides may foster a profound understanding, bridging the chasm between individual needs for recognition and institutional ecosystems that are bias- and stigma-free. Enhanced insight into the issue requires careful development of discourses, policies and interventions for suicide prevention. In this paper, we reconstruct the institutional responsibilization (Vincent et al., 2024) for work-related suicides, allowing reflexive policies, practices, and discourses to emerge for prevention.

## Conclusion

Durkheim originally identified four different rationales for suicide: Altruistic suicide happens when an individual sacrifices their life for the sake of others. Egoistic suicide happens when an individual decides to end their own life due to personal reasons. Fatalistic suicide happens when the individual sees no other option but to attempt suicide in a setting of rigid social regulation. Anomic suicides happen when social regulations are weak and social support to prevent suicides does not exist. In this paper, we focus on the case of anomie for suicides in the Turkish context, which has corroding social regulations due to the deregulatory pressures of neoliberalism. To address the gap we identified in institutional responsibility in work-related suicides and their prevention, we focused on anomie in work settings to see how the lack of institutional mechanisms to prevent suicides manifests as institutional suicides and identify mechanisms of institutional suicides, mobilizing concepts of lack of recognition and resultant dehumanization (Honneth, 2001; Haslam, 2006) and misrecognition through devaluation of varied form of human capital (Bourdieu, 1997). In the past, the state took on the role of overseeing and “normalizing” individuals dealing with mental health issues, while modern approaches prioritize self-care (Hennekam et al., 2023). Turkey faces similar circumstances, requiring proactive solutions for issues like inadequate mental health hospital infrastructure and a shortage of mental health professionals. Our study examines institutional responsibilization (Vincent et al., 2024) as a significant step toward preventing suicides. To achieve this, we turned the last wishes of suicide decedents into social policy design for prevention and suggested both individual and institutional responsibilization.

**Figure 1:**
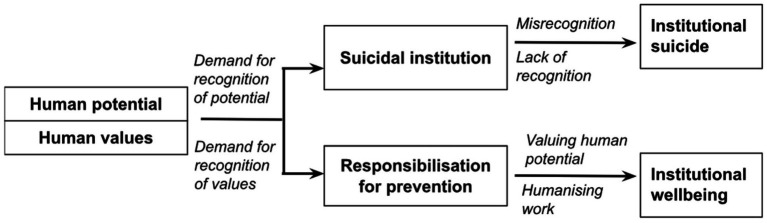
Antecedents, mechanisms of institutional suicide, and responsibilization for institutional wellbeing.

Drawing on suicide notes of decedents in work-related suicides, we identified two demands that they directed at their institutions. These demands are related to recognizing their human potential and values at work. We revealed two mechanisms of suicidal institutions. These are misrecognition of human potential and lack of recognition, which manifests as dehumanization. We identified two alternative strategies for preventing suicides through individual and institutional responsibilization. These are recognition of human potential, such as recognition of varied resources and capital individuals bring to work, and humanization of work through respect and compassion for workers. We argue that while suicidal institutions foreground institutional suicides, responsibilization for suicides may prevent suicides and foster institutional wellbeing (Özbilgin and Erbil, 2021). We outline our process model based on our findings in Figure 1.

Future research should examine understanding institutional suicides by extending its focus to diverse contexts and settings beyond Turkey, assessing the universality and variability of the identified mechanisms. Longitudinal studies are crucial to observe the long-term effects of institutional policies on suicide rates. It is essential to evaluate the effectiveness of various intervention strategies and co-design ideas in different institutional contexts to identify best practices for suicide prevention. Research should also explore the psychological and sociological dimensions of misrecognition and dehumanization within institutions, particularly how these phenomena manifest across various cultural and organizational settings. Comparative analyses between institutions with differing suicide rates could reveal key factors that contribute to healthier work environments. Moreover, the influence of national laws and policies on institutional behaviors and cultures, especially concerning suicide prevention, requires comprehensive examination to understand how legal frameworks can effectively motivate institutional change and accountability. Additionally, future research could investigate the background stories of the workplace and domestic violence about suicide, providing a more comprehensive understanding of the factors contributing to such tragic outcomes.

## Data availability statement

Publicly available information has been analyzed in this study. Links to the online sources have not been included with the article in order to protect the privacy of the individuals affected. Queries regarding the source material should be directed to the corresponding author.

## Ethics statement

The Ethics Committee of Ankara Haci Bayram Veli University confirmed that the study, which involved the analysis of publicly available material, was not subject to formal ethics or consent procedures in accordance with the local legislation and institutional requirements. Significant efforts were made to respect the sensitivity of the information, and protect the privacy of the individuals affected and their families and friends.

## Author contributions

MO: Writing – original draft, Writing – review & editing. CE: Writing – original draft, Writing – review & editing. OD: Writing – original draft, Writing – review & editing. NG: Writing – original draft, Writing – review & editing. KŞ: Writing – original draft, Writing – review & editing.
